# Burnout among Croatian physicians: a cross-sectional national survey

**DOI:** 10.3325/cmj.2019.60.255

**Published:** 2019-06

**Authors:** Vanja Pintarić Japec, Luka Vučemilo, Davor Kust, Alen Babacanli, Doris Dodig, Vesna Štefančić, Ksenija Vučur, Ana Brechelmacher, Matej Katavić, Krešimir Luetić, Tomislav Kopjar

**Affiliations:** 1 Health Center Zagreb – Center, Zagreb, Croatia; 2 Department of Otorhinolaryngology, University Hospital Merkur, Zagreb, Croatia; 3 Department of Oncology and Nuclear Medicine, Sestre milosrdnice University Hospital Center, Zagreb, Croatia; 4 Department of Emergency Medicine, Sestre milosrdnice University Hospital Center, Zagreb, Croatia; 5 Department of Radiology, University Hospital Center Rijeka, Rijeka, Croatia; 6 Croatian Institute of Public Health, Zagreb, Croatia; 7 Department of Nephrology, University Hospital Merkur, Zagreb, Croatia; 8 Department of Nephrology, University Hospital Dubrava, Zagreb, Croatia; 9 Department of Pediatrics, Sestre milosrdnice University Hospital Center, Zagreb, Croatia; 10 Department of Gastroenterology, University Hospital Sveti Duh, Zagreb, Croatia; 11 Department of Cardiac Surgery, University Hospital Center Zagreb, University of Zagreb School of Medicine, Croatia

## Abstract

**Aim:**

To investigate the prevalence of burnout syndrome among physicians of all specialties, including residents and non-specialists, on a national level in Croatia.

**Methods:**

This cross-sectional study, conducted in October 2017, used anonymous online survey based on the Maslach Burnout Inventory Human Services Survey. The Croatian version of the inventory was assessed for acceptability, factorial validity, and reliability. Key dimensions of burnout – emotional exhaustion, depersonalization, and lack of personal accomplishment were assessed. Respondents scoring high for emotional exhaustion or depersonalization were defined as burned-out.

**Results:**

The response rate was 18% (2557/14 427). Respondents’ median age was 41 years (range 25-80), and 68% (1737/2557) were women. Good sampling adequacy and scale reliability were confirmed. Factorial validity suggested the presence of three overall factors, and no items were eliminated. Sixty-three percent of physicians were burned-out. High score on emotional exhaustion, depersonalization, and reduced personal accomplishment were found in 58%, 29%, and 52% of respondents, respectively. As many as 16% of the respondents simultaneously experienced high levels of all three burnout dimensions. Multivariate logistic regression analysis revealed that residents and physicians in tertiary or primary care were at an increased risk of burnout, while physicians working in institutes were at a decreased risk.

**Conclusion:**

Active national measures are needed to reduce the high prevalence of burnout among Croatian physicians.

Chronic workplace stressors can lead to burnout, a psychological syndrome characterized by three key dimensions – emotional exhaustion (EE), depersonalization (DP), and feelings of reduced personal accomplishment (PA). EE refers to feelings of being overextended and depleted of one's emotional and physical resources, DP represents an excessively detached response to various job aspects, while lack of PA refers to feelings of job-related incompetence and lack of achievement (1). The Maslach Burnout Inventory (MBI) has been recognized as the gold standard measurement tool for burnout (2).

Burnout is etiologically, clinically, and nosologically similar to depression (2). Burned-out physicians can have physical and psychological symptoms of burnout (3). Burnout can occur at any age, already during residency (4), and can lead to poor quality of patient care and medical errors (3). It is associated with decreased productivity and leaving a working position or even the entire field of medicine (5). Furthermore, it can also be associated with young physicians’ wish to emigrate (6). Since it can clearly have deleterious effects on physicians, patient care, and the entire health care system, active preventive measures are necessary (7).

Causes and predictors of burnout are various and linked not only to workload. According to several former studies, stressors in health care workers can be both environmental and individual. Some of the important ones are intrinsic factors of work, administration, stressors related to financial opportunities, contact with patients, relationships with coworkers, organizational structure and climate, interference of private and work life, and role ambiguity (8,9).

Although nationwide burnout syndrome studies among physicians have been conducted in Europe, these studies only included specific physician groups, such as solely family physicians or residents (10-12). Furthermore, a large international study of burnout syndrome conducted in twelve European countries involved only family physicians (13). To the best of our knowledge, there is no national study among European countries investigating burnout syndrome involving physicians of all specialties, all levels of health care system, working both in the private and public sector.

The Croatian health care system experiences a deficit of human resources, with approximately 4% of all Croatian physicians having gone to work abroad in a three-year period (2013-2016), leaving the remaining personnel with an increasing workload (14). The aim of this study is to investigate the prevalence of burnout syndrome among physicians in Croatia on a national level and in different specialty groups.

## METHODS

### Study design

This cross-sectional study was conducted through an online survey, which was freely available for participation between October 17 and October 27 in 2017. The survey was completely anonymous. Data were collected through an online Google Forms platform. The study inclusion criteria were set to include physicians practicing in the Croatian health care system, from both public and private sector, and regardless of their age, specialty, geographical region, level of health care, title or working status. The only exclusion criterion was practicing outside of Croatia.

An e-mail invitation to participate in the survey with dedicated instructions was sent to the members of the Croatian Medical Chamber, a regulatory body of physicians practicing in Croatia with mandatory membership. The email also included a cover letter for the physicians, asking for their voluntary participation, explaining the purpose of the research, and providing a hyperlink to the survey. One reminder email was sent five days after the initial invitation and the survey was closed five days subsequently. This study was approved by the Committee for Medical Ethics and Deontology of the Croatian Medical Chamber (class: 030-02/18-11/55, number: 385-02-03/02-18-02, date: April 16, 2018).

### Instrument and scoring

The survey was based on the Croatian version of the MBI Human Services Survey (HSS) (15). The instrument licenses and scoring key, as well as the approval for remote online use, were obtained from Mind Garden, Inc. (Menlo Park, CA, USA). We adapted the inventory for our target population replacing the word “*korisnik*” (Croatian for recipient) with the word “*pacijent*” (Croatian for patient). This is a common practice since this survey is intended to be used by people in a wide variety of occupations. The MBI – HSS includes 22 items on a 7-point Likert-type frequency scale (0 – never; 1 – a few times a year or less; 2 – once a month or less; 3 – a few times a month; 4 – once a week; 5 – a few times a week; 6 – every day). The 22-item assessment evaluates and scores three burnout dimensions: EE, DP, and lack of PA. Scores are then categorized based on the provided scoring key as low level (EE<17, DP<7, PA>38), moderate level (EE 17-26, DP 7-12, PA 32-38), or high level (EE>26, DP>12, PA<32). PA scoring is inversed in order to measure the lack of PA. Except for the questions of the MBI – HSS, some demographic descriptors were also collected. Demography questionnaire included participant age, sex, marital status, number of children, professional title, specialty, academic title, country of practice, institution of employment, length of employment, and county of practice.

EE and DP have been suggested to be the foundation of burnout (15). Participants scoring high for EE or DP dimensions were defined as burned-out. Therefore, the participants were grouped either in the burned-out or in the non-burned-out group. Our demographic questionnaire defined the working environment as primary, secondary, tertiary care, institutes, and other based on the level of care provided by the institution of employment. Specialists, subspecialists, and physicians in training were grouped according to their specialty field into three categories surgical, non-surgical, and diagnostic and public health (Supplementary Table 1(supplementary Table 1)).

### Statistical analysis

Data were summarized by using standard descriptive statistics. Normality of distribution of continuous variables was tested by the Shapiro-Wilk test and Quantile-Quantile Plots. The continuous data are presented as mean ± standard deviations or medians with interquartile range (IQR) or range, where appropriate. Categorical variables are presented as absolute numbers and percentages. Difference between participants with and without burnout was evaluated using the independent-samples Mann-Whitney U test (for continuous variables) or χ^2^ test (for categorical variables). All statistical tests were two-sided. The significance level was set at *P* < 0.05.

Multivariate binary logistic regression analysis was performed to identify burnout risk factors. We selected the potential risk factors for the multivariate analysis based on the variables available in the single factor analysis of respondents with and without burnout. The variables selected for the multivariate analysis were age, sex, marital status, number of children, title, specialty, length of employment, working environment, and academic title. To establish the logistic regression model, we used a backward algorithm. The criteria for entry and removal from the model at each step were set at *P* < 0.05 and *P* > 0.1, respectively. In order to control for age and sex, the two variables were forced into the final model after the backward elimination process. Adjusted odds ratios (OR) and 95% confidence intervals (CI) were calculated for the variables in the equation.

We validated the inventory for the population of Croatian physicians. An exploratory factor analysis was used to uncover any latent variables that cause the manifest variables to covary in the Croatian version of the MBI – HSS questionnaire. During factor extraction, the questionnaire items are grouped depending on the variance they share, which is captured by factors that are interpreted as latent dimensions. To assess for sampling adequacy, we ran the Kaiser-Meyer-Olkin test. The suitability of the item correlation matrix for factoring was tested with the Bartlett’s test of sphericity. In exploratory factor analysis we used the maximum likelihood estimation with direct oblimin rotation. We included the extracted factors with the eigenvalue >1, which accounted for more >10% of the variance, and which passed the visual inspection on the scree plot. Cronbach’s alpha with 95% CI was used to assess internal consistency, a measure of scale reliability. Statistical analyses were performed using the open-source R software, version 3.5.1 (R Foundation for Statistical Computing, Vienna, Austria).

## RESULTS

### Participants

**Figure 1:**
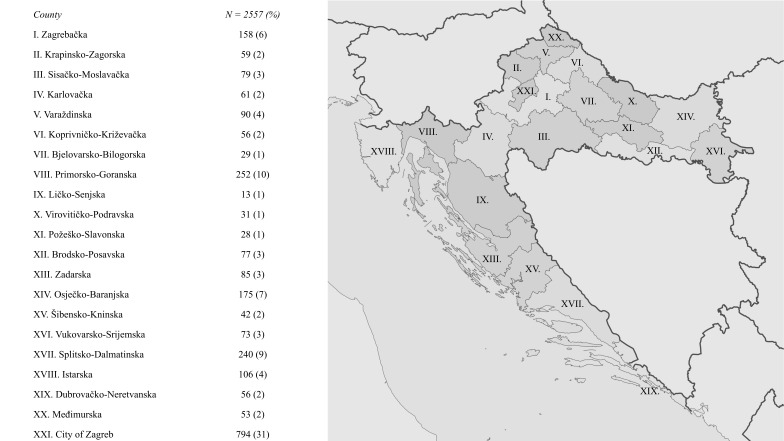
Geographical distribution of respondents.

There were 2557 respondents eligible for the study. Median age of respondents was 41 years (range, 25-80; IQR, 33-52) and 68% (n = 1737) were women. The median length of service was 15 years (IQR, 7-25.5). About two thirds of respondents had completed a training program (62%, n = 1594), were married (63%, n = 1620), and had children (66%, n = 1684). Likewise, 66% (n = 1697) worked in either secondary or tertiary care institutions. The most common specialty was family medicine (Supplementary Table 1(supplementary Table 1)). Respondents working in tertiary hospitals formed the largest group (37%, n = 954). About a half of the survey sample (48%, n = 1219) comprised the respondents of non-surgical specialty (Table 1). The survey involved physicians from each of the 21 Croatian counties (Figure 1).

**Table 1:** Participants’ demographic characteristics (N = 2557)

Characteristics	Number (%)
Age (years)	
<30	353 (14)
30-39	808 (32)
40-49	584 (23)
50-59	603 (24)
≥60	209 (8)
Sex	
male	820 (32)
female	1737 (68)
Marital status	
single	574 (22)
married	1620 (63)
divorced	149 (6)
domestic partnership	214 (8)
Number of children	
without children	873 (34)
1 child	587 (23)
2 children	842 (33)
>2 children	255 (10)
Title	
no specialty	358 (14)
resident	605 (24)
specialist	1058 (41)
subspecialist	536 (21)
Specialties	
surgical	616 (24)
non-surgical	1219 (48)
diagnostic	322 (13)
not specified	42 (2)
no specialty	358 (14)
Length of employment (year)	
0-10 y	956 (37)
11-20 y	722 (28)
21-30 y	503 (20)
31-40 y	376 (15)
Working environment	
primary care	482 (19)
secondary care	743 (29)
tertiary care	954 (37)
institutes	233 (9)
other	145 (6)
Academic title	
no title	2014 (79)
chief physician	61 (2)
master’s degree	104 (4)
doctor’s degree	218 (9)
associate professor	92 (4)
professor	68 (3)

### Response rate

During the 10-day period when the survey was online, 2568 participants completed the questionnaire. Out of the 2568 completed questionnaires, 11 were excluded since the respondents were practicing abroad. Finally, 2557 questionnaires were included in the analysis. Based on the data from the 2016 Croatian Health Statistics Yearbook, there were 14427 physicians working in the Croatian health care in 2016 (16). The response rate of the survey was, therefore, 18% (2557/14427).

### Validation

The data were screened for multivariate assumptions. There were no missing data, and the subject-to-item ratio was >100:1. Good correlation and sampling adequacies were confirmed. Bartlett’s test indicated correlation adequacy with a *P* < 0.001, while the Kaiser-Meyer-Olkin test indicated sampling adequacy, with the overall measure of sampling adequacy index of 0.91.

A parallel principal component and factor analysis, and scree plot examination (Supplementary Figure 1(supplementary Figure 1)) suggested three overall factors/components in the Croatian version of the MBI – HSS questionnaire, and a three-factor model was tested on theory. These three factors account for 54% of cumulative variance. After testing all 22 questions, no items split across all three factors using the criterion that loadings must be >0.3 (Supplementary Table 2(supplementary Table 2)), and no items were eliminated. The root mean square error approximation was 0.08 (90% CI, 0.07-0.08) and the comparative fit index was 0.92, both indicating a well-fitted model.

The overall Cronbach’s alpha value for the MBI – HSS was 0.84 (0.84-0.85). For the EE dimension it was 0.70 (0.68-0.72), for DP – 0.83 (0.82-0.84), and for the lack of PA – 0.92 (0.91-0.92). All values were ≥0.7, indicating good scale reliability.

### Burnout

**Figure 2:**
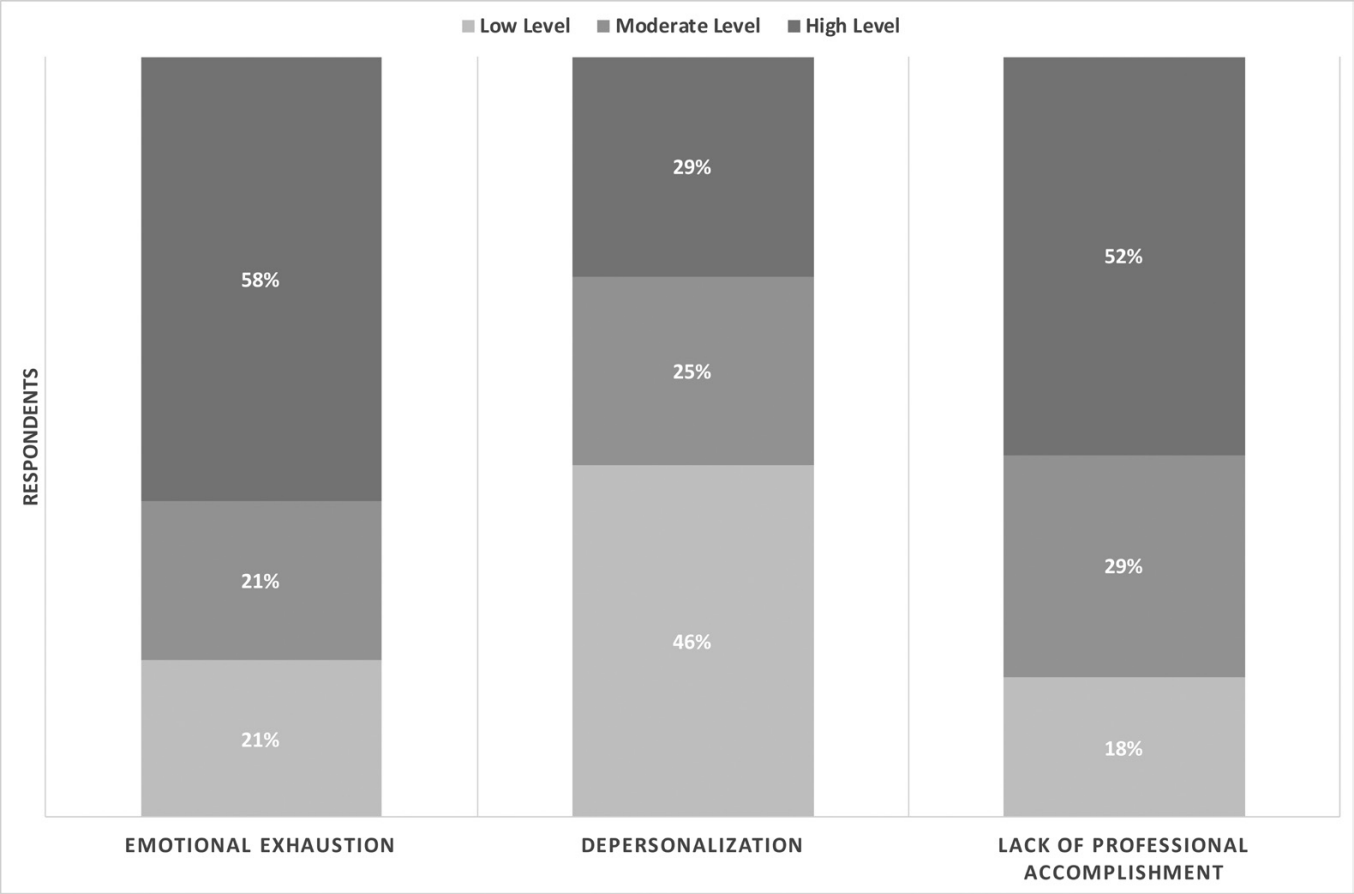
The prevalence of three burnout dimensions in 2557 respondents.

The means for EE and lack of PA scores were in the high level range, while the mean for DP score was in the moderate level range (Table 2). A total of 35% of respondents (n = 628) had a high score on both EE and DP levels, while 33% (n = 856) had a high score on both EE and lack of PA levels. As many as 16% (n = 421) of the respondents simultaneously experienced high levels of all three burnout dimensions. According to the proposed definition of a burned-out respondent – high levels of either EE or DP, 63% (n = 1604) of physicians were categorized as burned-out (Figure 2).

**Table 2:** Burnout among responding physicians (N = 2557)

**Emotional exhaustion**	
Score, mean ± standard deviation	29 ± 13
Score level, n (%)	
low (<17)	528 (21)
moderate (17-26)	535 (21)
high (>26)	1494 (58)
Depersonalization	
Score, mean ± standard deviation	9 ± 7
Score level, n (%)	
low (<7)	1182 (46)
moderate (7-12)	637 (25)
high (>12)	738 (29)
Lack of personal accomplishment	
Score, mean ± standard deviation	30 ± 9
Score level, n (%)	
low (>38)	470 (18)
moderate (32-38)	749 (29)
high (<32)	1338 (52)
Burned-out, n (%)*	1604 (63)

*High score on the emotional exhaustion or depersonalization subscale.

### Comparison of burned-out and non-burnout-out respondents

There was no significant difference between the groups in age, sex, marital status, and number of children, while there were significant differences in title, specialties, working environment, and academic title. The frequency of burnout was higher among respondents in training (residents), those with non-surgical specialties, and those working in tertiary hospitals. The frequency of burnout was lower among respondents working in institutes and those with a master’s degree (Table 3).

**Table 3:** Comparison of demographic characteristics of burned-out and non-burned-out respondents*

	Burned-out (n = 1604)	Non-burned-out (n = 953)	*P*
Age (year)	42 (33-53)	41 (33-52)	0.822
<30	225 (14)	128 (13)	0.673
30-39	503 (31)	305 (32)	0.734
40-49	359 (22)	225 (24)	0.474
50-59	388 (24)	215 (23)	0.348
≥60	129 (8)	80 (8)	0.753
Sex			
female	1083 (68)	654 (69)	0.562
male	521 (32)	299 (31)	0.562
Marital status			
single	360 (22)	214 (22)	0.995
married	1015 (63)	605 (63)	0.917
divorced	97 (6)	52 (5)	0.537
domestic partnership	132 (8)	82 (9)	0.741
Number of children	1 (0-2)	1 (0-2)	0.763
without children	549 (34)	324 (34)	0.906
1 child	366 (23)	221 (23)	0.829
2 children	518 (32)	324 (34)	0.375
>2 children	171 (11)	84 (9)	0.132
Title			
no specialty	208 (13)	150 (16)	0.051
resident	403 (25)	202 (21)	0.024
specialist	665 (41)	393 (41)	0.913
subspecialist	328 (20)	208 (22)	0.408
Specialties			
surgical	375 (23)	241 (25)	0.275
non-surgical	789 (49)	430 (45)	0.046
diagnostic	201 (13)	121 (13)	0.903
not specified	31 (2)	11 (1)	0.134
Length of employment (years)	15 (6-26)	15 (7-25)	0.737
0-10 y	605 (38)	351 (37)	0.654
11-20 y	434 (27)	288 (30)	0.086
21-30 y	318 (20)	185 (19)	0.799
31-40 y	247 (15)	129 (14)	0.198
Working environment			
primary care	308 (19)	174 (18)	0.555
secondary care	452 (28)	291 (31)	0.205
tertiary care	642 (40)	312 (33)	<0.001
institutes	115 (7)	118 (12)	<0.001
other	87 (5)	58 (6)	0.484
Academic title			
no academic title	1281 (80)	733 (77)	0.078
chief physician	40 (2)	21 (2)	0.642
master’s degree	52 (3)	52 (5)	0.006
doctor’s degree	139 (9)	79 (8)	0.742
associate professor	55 (3)	37 (4)	0.552
professor	37 (2)	31 (3)	0.150

*Data are presented as n (%) or median (interquartile range).

Some of the variables that were shown to be significantly different in univariate analysis did not show the same tendency in the multivariate analysis. The variable selection for the multivariate analysis was based on the comparison of respondents with and without burnout. The multivariate logistic regression model showed the risk factors for burnout. The model was significant and the Cox & Snell’s and Nagelkerke’s R^2^ were 0.022 and 0.030 respectively. Physicians in training (residents) and those in tertiary or primary care working environment were at an increased risk of burnout, as well as those without an academic title or holding a doctorate (Table 4). On the other hand, physicians working in institutes were at a decreased risk of burnout.

**Table 4:** Risk factors for burnout (multivariate binary logistic regression analysis)

	Odds ratio (95% confidence interval)	*P*
Age	1.006 (0.997-1.015)	0.195
Female	0.97 (0.81-1.15)	0.704
No specialty	0.75 (0.55-1.01)	0.062
Resident	1.39 (1.08-1.78)	0.01
Primary care	1.42 (1.08-1.88)	0.012
Tertiary care	1.43 (1.17-1.75)	<0.001
Institutes	0.61 (0.46-0.82)	0.001
No academic title	1.68 (1.29-2.2)	<0.001
Doctor’s degree	1.48 (1.02-2.15)	0.038
Chief physician	1.64 (0.91-2.96)	0.096

## DISCUSSION

The MBI – HSS questionnaire revealed that the two crucial dimensions for the definition of burnout, EE or DP, were high in 63% of the respondents. This confirms that burnout is common in the population of Croatian physicians. Although the analysis did not identify any one particular specialty group to be at risk of burnout, physicians in training and those in tertiary or primary institutions, as well as those without an academic title and those holding a doctorate, were found to be at risk.

Since the questionnaire used was not previously validated for the population of Croatian physicians, we assessed its validity and reliability. The validation methods used deemed the Croatian version of the MBI – HSS questionnaire suitable for the population of Croatian physicians. We acknowledge that the reliability of the EE dimension is not very high, since the confidence interval spans below the boundary margin. However, the alpha coefficient for all three dimensions was 0.84, suggesting relatively high overall internal consistency. Exploratory factor analysis grouped the items of the MBI – HSS questionnaire into three latent dimensions. The three extracted factors correspond to the three key dimensions of burnout assessment. None of the items of the inventory split across all three extracted factors with high loadings and therefore no item was discarded.

Several studies focused only on EE and DP among physicians (17,18) and considered that high scores in these dimensions indicated a burned-out professional (19,20). In our study almost two thirds of physicians were categorized as burned-out, suggesting that burnout among Croatian physicians is at a worrisome level. Our results are consistent with earlier reports in which burnout affected a great proportion of physicians and residents, often leading to devastating personal and professional consequences, depression, increased stress levels, and overall lower quality of life (7,18,21). In comparison with other European studies (13,22), our respondents showed higher levels of burnout on EE and PA subscales, while DP rates were comparable. However, since other research has shown that accumulated effects of EE can lead to long-term erosion of physician’s idealism and DP symptoms (23), it might be a matter of time when DP levels among Croatian physicians will follow the other two subscales. Marcelino et al (10) showed significantly lower burnout levels among Portuguese family physicians; high EE levels were present in 25% of respondents, high DP levels in 16%, and high lack of PA subscale levels in 17% of respondents, while only 2% of respondents showed high burnout levels in all three categories. In our study, as many as 16% of respondents simultaneously experienced high levels on all three burnout dimensions.

Burnout among Croatian physicians was previously investigated in two subpopulations of physicians: in a tertiary hospital environment at the University Hospital Center Rijeka (24) and among family doctors through the Croatian arm of the European General Practice Research Network (25). According to Tomljenović et al (24), 44%, 34%, and 49% of respondents from the University Hospital Center Rijeka reported high burnout scores of EE, DP, and lack of PA domains, respectively. In the study by Ožvačić et al, 42%, 16%, and 15% Croatian family physicians reported high levels of EE, DP, and lack of PA, respectively (25). Our results showed higher levels of EE and lack of PA than both of these studies; while DP levels were similar to Tomljenović et al they were still higher than those reported by Ožvačić et al (24,25). Furthermore, an international survey in seven European countries showed lower proportion of high EE and DP levels (EE = 21% and DP = 19%) among Croatian health professionals than our study (26). However, only 31% (N = 60) of the Croatian participants were physicians, and the data were collected 5 years before our study (26). A US study showed that burnout levels among physicians gradually worsen (21), which might explain the difference between our results and earlier data from Croatia. Moreover, our study included front-line caregivers, such as emergency medicine, primary care, and general internal medicine physicians, which were shown to be at an increased risk of burnout (27).

Even though among demographic characteristics we did not find independent risk factors for burnout, the risk was increased in residents. These results are in line with a study that confirmed residents to be especially prone to burnout (28). Contrary to this, a recent study from Slovenia investigating burnout syndrome among family physicians found higher levels of burnout among specialists compared with residents (29). However, these results could be attributed to the sampling method and the specificities of the Slovenian health care system (29). Residency training is a period often marked by a lack of autonomy, high educational demands, and long working hours (12). Furthermore, there is strong evidence that a positive learning environment with appropriate mentorship is of key importance in preventing resident burnout (12,30). Well organized mentorship programs could alleviate high levels of stress and burnout within surgical residency programs and achieve high levels of personal satisfaction as well as an improved overall quality of life (31). Our recent study of factors influencing young physicians’ dissatisfaction in Croatia showed that residents were greatly dissatisfied with the role mentors played in their training and were burdened by pointless administrative tasks during their residency programs (32). Considering all this evidence, we postulate there is room for improvement and prevention of further resident burnout among Croatian physicians by advancing residency and mentorship programs.

When exploring the effect of working environment on burnout we found that physicians working in tertiary hospitals were mostly affected and at the greatest risk, followed by those working in primary care. The study on prospective predictors of professional burnout in hospital nurses of University Hospital Center Rijeka showed that EE and DP were most significantly influenced by role conflict and work overload (9). Based on the data available in the Demographic Atlas of Croatian Practicing Physicians, physicians working in secondary care had the highest workload, followed by tertiary care physicians, while those working in institutes or primary care had significantly lower workloads (14). This might be due to lack of practicing physicians in secondary care institutions (14). At both secondary and tertiary health care levels in Croatia, physicians work 24-hour shifts, have similar material rights and financial resources in terms of stringent hospital supplies and infrastructure. We assume that the burnout among physicians working at tertiary care institutions is related to higher complexity of care, severely ill and demanding patients, and additional pressure caused by educational and scientific activities, which are attributes specific to the tertiary care environment in Croatia.

While Tomljenović et al did not find any difference between surgical, non-surgical, and diagnostic groups of respondents (24), in our study non-surgical group experienced more burnout than the remaining groups. However, multivariate analysis did not show non-surgical specialty to be an independent risk factor. This finding could be concordant to the research that showed front-line care specialties such as emergency, family, and internal medicine to be at the highest risk (22,27). Surgical medical professionals have the highest workload compared with other specialties in Croatia (14). Since we did not find a particular specialty group to be at risk of burnout, we assume that burnout syndrome among Croatian physicians is not only a consequence of high workload, but rather associated with factors related to the working environment.

An important strength of our study is the cross-sectional design, with a good response rate for an online survey (33), as well as a fair representation of all age groups, specialties, geographical territories, levels of health care, and working status. The main limitation of our study is the use of an anonymous online survey and lack of control for multiple entries from the same participant. Compared with the demographic data of Croatian physicians (14), a higher proportion of women (68% vs 63%) and lower mean age of the respondents (43 vs 46) indicate the potential for sampling error and selection bias. Our sample might not fully represent the entire population of physicians in Croatia and may be biased toward physicians dissatisfied with their working conditions. Indeed, those not experiencing burnout may have elected not to respond. Furthermore, we did not investigate the consequences of burnout and we did not investigate the impact of personality traits as one of the predictors of burnout (9).

Other studies have shown that burnout syndrome can lead to physicians wishing to leave their current work position or the entire field of medicine (5). The Croatian health care system experiences a shortage of physicians, since numerous physicians left the country to work abroad. From 2013 until 2016, 525 physicians, approximately 4% of all Croatian physicians left the Croatian public health system (14). This contributes to an even higher workload for the remaining physicians, manifested by an 11% increase in overtime working hours in 2017, based on the data from the Croatian Financial Agency (personal communication). Further research into this issue is needed. Policy makers and health care organizers should consider the high prevalence of burnout among Croatian physicians and its potential effect on the quality of care, migration of physicians, and the sustainability of the entire health care system.
