# Supplementary material for: Burnout among Croatian physicians: a cross-sectional national survey
Source: Croat Med J. 2019 Jun;60(3):255–64. doi: 10.3325/cmj.2019.60.255 (PMC6563170; doi:10.3325/cmj.2019.60.255)
Supplement: Supplementary Table 1 [file CroatMedJ_60_s003.pdf]

Supplementary Table 1. Specialties Classified in Groups.

	N = 2557 (%)
Non-surgical	*1219 (48)*
Family medicine	283 (11)
Paediatrics	178 (7)
Psychiatry	117 (5)
Emergency medicine	79 (3)
Neurology	77 (3)
Cardiology	74 (3)
Internal medicine	52 (2)
Physical medicine and rehabilitation	51 (2)
Oncology and radiotherapy	47 (2)
Gastroenterology	37 (1)
Oncology	36 (1)
Nephrology	36 (1)
Endocrinology and diabetology	31 (1)
Pulmonology	30 (1)
Dermatology and venerology	27 (1)
Infectious diseases	24 (0.9)
Haematology	17 (0.7)
Rheumatology	9 (0.4)
Allergology and clinical immunology	6 (0.2)
Paediatric and adolescent psychiatry	6 (0.2)
Clinical pharmacology and toxicology	1 (0.04)
Paediatric infectious diseases	1 (0.04)
Surgical	*616 (24)*

Anaesthesiology	170 (7)
Obstetrics and gynaecology	116 (5)
Ophthalmology	69 (3)
Orthopaedics and traumatology	60 (2)
Otorhinolaryngology	46 (2)
General surgery	41 (2)
Urology	35 (1)
Abdominal surgery	34 (1)
Paediatric surgery	11 (0.4)
Neurosurgery	9 (0.4)
Vascular surgery	8 (0.3)
Maxillofacial surgery	7 (0.3)
Cardiothoracic surgery	5 (0.2)
Plastic and reconstructive surgery	5 (0.2)
No speciality	*358 (14)*
Diagnostic	*322 (13)*
Clinical radiology	95 (4)
Occupational and sports medicine	46 (2)
School and adolescent medicine	45 (2)
Pathology and cytology	39 (2)
Epidemiology	28 (1)
Nuclear medicine	19 (0.7)
Transfusion medicine	19 (0.7)
Clinical microbiology	16 (0.6)
Public health	13 (0.5)
Laboratory medicine	1 (0.04)

Forensic medicine	1 (0.04)
Not specified	*42 (2)*
