# Supplementary figures and images for: Burnout among Croatian physicians: a cross-sectional national survey
Source: Croat Med J. 2019 Jun;60(3):255–64. doi: 10.3325/cmj.2019.60.255 (PMC6563170; doi:10.3325/cmj.2019.60.255)

Supplementary Figure 1.

Parallel Analysis Scree Plots

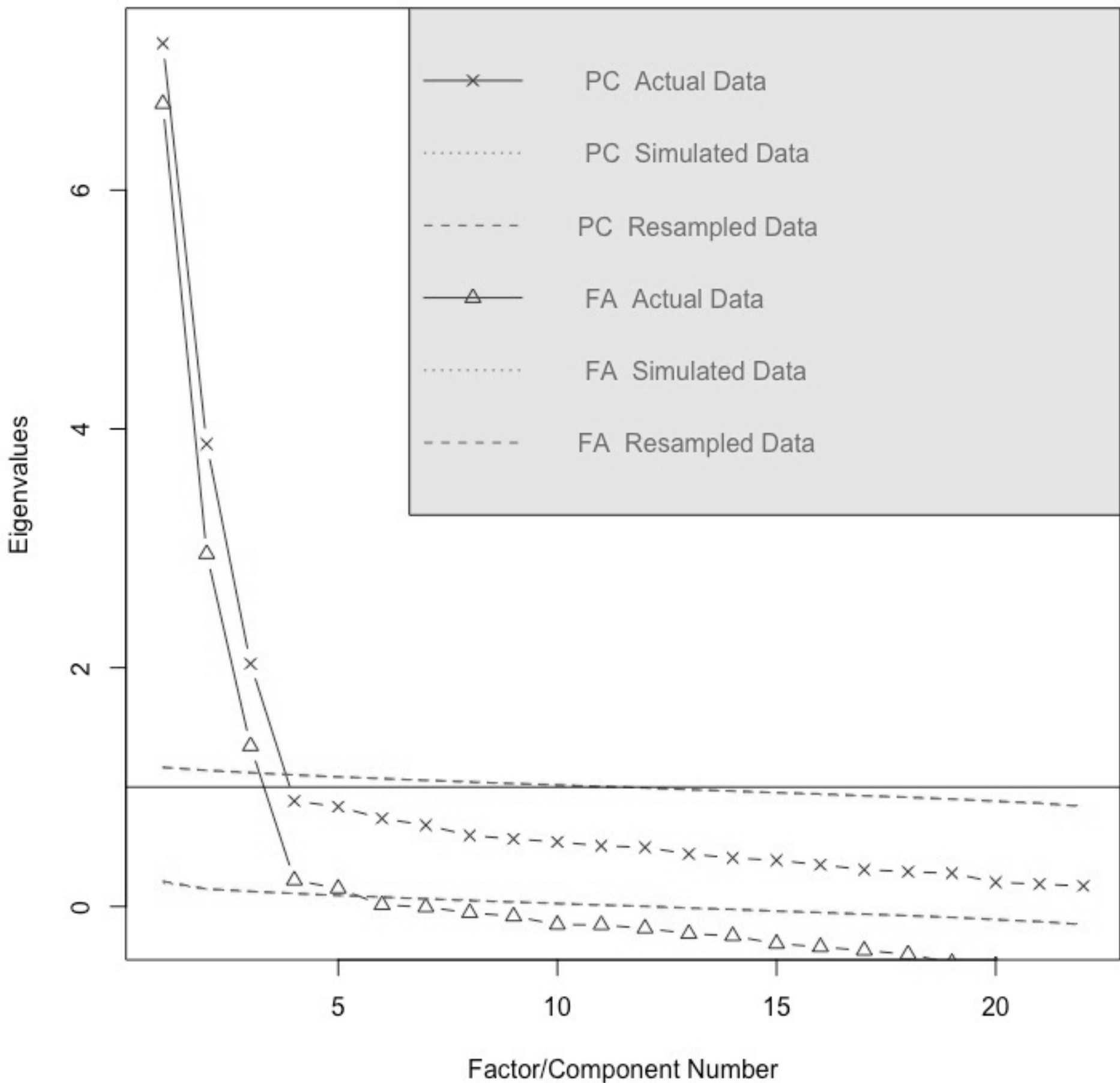

Supplement: Supplementary Figure 1 [file CroatMedJ_60_s004.pdf]
