# Supplementary material for: Burnout among Croatian physicians: a cross-sectional national survey
Source: Croat Med J. 2019 Jun;60(3):255–64. doi: 10.3325/cmj.2019.60.255 (PMC6563170; doi:10.3325/cmj.2019.60.255)
Supplement: Supplementary Table 2 [file CroatMedJ_60_s005.pdf]

Supplementary Table 2. Factor matrix with loadings for each item of the Maslach Burnout Inventory Human Services Survey

	**Emotional Exhaustion**	**Personal Accomplishment**	**Depersonalization**
Q01 - EE	0.88	0.02	-0.04
Q02 - EE	0.88	0.07	-0.03
Q03 - EE	0.76	-0.02	0.05
Q04 - PA	0.33	0.49	-0.08
Q05 - DP	0.1	-0.02	0.62
Q06 - EE	0.51	-0.06	0.31
Q07 - PA	0.06	0.65	0.06
Q08 - EE	0.86	-0.03	0.02
Q09 - PA	0.04	0.68	-0.02
Q10 - DP	-0.02	-0.01	0.91
Q11 - DP	0.03	0.01	0.83
Q12 - PA	-0.48	0.57	0.12
Q13 - EE	0.68	-0.04	0.16
Q14 - EE	0.76	0.13	0.01
Q15 - DP	0	-0.03	0.61
Q16 - EE	0.45	-0.05	0.25
Q17 - PA	0.06	0.75	-0.03
Q18 - PA	-0.04	0.7	-0.07
Q19 - PA	0.08	0.59	-0.07
Q20 - EE	0.82	-0.05	0.03
Q21 - PA	-0.06	0.62	0.03
Q22 - DP	0.18	0.01	0.35

EE: Emotional Exhaustion; DP: Depersonalization; PA: personal Accomplishment
